# Calculating the dose of cisplatin that is actually utilized in hyperthermic intraperitoneal chemotherapy among ovarian cancer patients

**DOI:** 10.1186/s13048-021-00764-6

**Published:** 2021-01-08

**Authors:** Wu-yun Wang, Miao-fang Wu, Dong-bing Wu, Li-juan Wang, Hui Li, Zhong-qiu Lin, Jing Li

**Affiliations:** 1 Department of Gynecologic Oncology, Sun Yat-sen Memorial Hospital, Sun Yat-sen University, Guangzhou, 510120 People’s Republic of China; 2 Department of Obstetrics and Gynecology, Kiang Wu Hospital, 85-87 R. de Coelho do Amaral, Macau, 96000 People’s Republic of China

**Keywords:** Ovarian cancer, Cisplatin, Hyperthermic intraperitoneal chemotherapy, Dose

## Abstract

**Background:**

Hyperthermic intraperitoneal chemotherapy (HIPEC) is an important treatment for ovarian cancer. A certain portion of cisplatin exits the body via the perfusate at the end of HIPEC, so full-dose utilization cannot be achieved. Herein, we sought to explore how much cisplatin is actually utilized and its prognostic influence.

**Methods:**

Cisplatin (70 mg/m^2^) was given at 43 °C for 90 min. The actually utilized dose (AD) of cisplatin was calculated using the following formula: AD (mg) = total dose (TD) (mg)-losing dose (LD) (mg); LD = volume (ml) of the perfusate (VP_retained_) that was retained in the HIPEC treatment system at the end of HIPEC * concentration of cisplatin in the perfusate (mg/ml).

**Result:**

Sixty-two ovarian cancer patients were included. The median TD, median LD and median AD were 95 mg, 20.7 mg and 75.8 mg, respectively. The utility rate of cisplatin (AD/TD ratio) was 79.2%. On simple linear regression analysis, the TD and VP_retained_ were found to significantly predict the AD. Based on these two factors, multiple linear regression analysis was conducted, and a significant regression equation was formulated [F (2, 59) = 71.419, *P* < 0.0001]: predicted AD (mg) = 30.079 + 0.667 TD (mg) – 0.010 VP_retained_ (ml) (adjusted *R*^2^ = 0.698). In Cox regression analysis, AD was not noted to be associated with progression free survival or overall survival.

**Conclusion:**

For ovarian cancer patients who receive cisplatin for HIPEC at 43 °C, the AD of cisplatin can be predicted using a regression equation and it has no prognostic impact.

**Supplementary Information:**

The online version contains supplementary material available at 10.1186/s13048-021-00764-6.

## Introduction

Ovarian cancer is the deadliest gynecologic cancer and is usually diagnosed after the cancer has spread beyond the ovary [1]. Primary debulking surgery (PDS) combined with chemotherapy has been the first-line treatment for ovarian cancer patients. However, even among patients without evidence of disease following treatment, 70% experience recurrence within the subsequent 3 years, and almost 80% succumb to their disease [2].

Because of a clear tendency to develop peritoneal metastasis, attempts to improve outcomes for this patient population have prompted the investigation of intraperitoneal chemotherapy (IPC), and its effect has been validated [3]. IPC can also be delivered under hyperthermic conditions, which is termed hyperthermic intraperitoneal chemotherapy (HIPEC). An increasing number of studies have shown a survival benefit associated with HIPEC among ovarian cancer patients [4–8]. Based on this evidence, HIPEC has been recommended in the National Comprehensive Cancer Network (NCCN) guidelines for patients receiving interval debulking surgery (IDS) [9]. In the China Anti-Cancer Association (CACA) guidelines, HIPEC is recommended as an adjuvant treatment for gynecologic cancer patients with peritoneal carcinomatosis; currently, it has been a publicly approved therapy in China and its costs are covered by insurance plans [10].

Owing to a favorable peritoneal plasma gradient, cisplatin is the preferred agent for IPC [11]. The addition of hyperthermia to intraperitoneal cisplatin can result in synergistic effects, thereby enhancing cytotoxicity [12]. Therefore, cisplatin is also the most commonly used drug in HIPEC [13]. A noteworthy observation in clinical practice is that a certain portion of cisplatin exits the body via the perfusate at the end of treatment, so full cisplatin uptake cannot be achieved. Previous investigations have explored the pharmacokinetic features of cisplatin in HIPEC [14–16]. However, no investigator could provide a clear-cut answer to the following critical question: how much cisplatin is actually utilized by a patient during HIPEC? Accordingly, it is unclear whether the amount of cisplatin depletion adversely affects therapeutic effect. We herein conducted a study to predict the actual uptake dose of cisplatin based on clinical variables that are easy to obtain and investigate its prognostic effect. From a clinical perspective, we believe that our findings are conducive to optimizing the dose planning and schedule of cisplatin in HIPEC.

## Materials and methods

After Institutional Review Board approval was obtained (Approval No. SYSEC-KY-KS-2020-046), a retrospective chart review was conducted. Ovarian cancer patients who received cisplatin for HIPEC between 2016 and 2018 were identified. Patients who were considered for HIPEC underwent detailed counseling with regard to the potential risks and benefits within the context of previously published clinical trials and guidelines. All patients provided signed informed consent. Data from patients who received intravenous platinum within 2 weeks before HIPEC and patients who did not undergo the complete HIPEC procedure were excluded.

Surgical complexity of the debulking surgery was based on a complexity score which was calculated using a published scoring system [17]. At the end of cytoreduction, four tubes were placed (two in the bilateral subdiaphragmatic space for use as inlet tubes and two in the pelvic cavity for use as an outlet tubes) before closing the incision, which were used to administrate HIPEC. HIPEC was given following surgery using a close technique. A high-precision hyperthermic intraperitoneal perfusion treatment system with a precision of ±0.10 °C for temperature control and ± 5% for flow control (approved by the State Food Drug Administration of China, approval no. 2009–3,260,924) was utilized. Cisplatin was given at a dose of 70 mg per square meter and was added to 3000–5000 ml of saline solution. The perfusate was heated and circulated at a flow rate of 300–500 ml/min. The perfusion velocity was adjusted to ensure that the entire abdomen was exposed to the perfusate (the initial velocity was 300 ml/min and increased gradually until the patient felt floated or until a flow rate of 500 ml/min was achieved). An intraabdominal temperature of 43 °C was maintained and measured by the treatment system using temperature monitoring probes in the infusion and outflow catheters. The HIPEC procedure took 90 min in total, consisting of a 30 min preheating period and a 60 min perfusion period. All patients received continuous intravenous fluids to assure adequate hydration. During the treatment, vital signs and urine output were monitored continually. After HIPEC treatment, the perfusate retained in the treatment system and in the tubes was collected after removing the tubes, and then the volume was measured. Thereafter, a portion of the perfusate was stored at − 80 °C and analyzed within 3 weeks. Perfusate collecting and volume assessment have been integrated into routine clinical care in our institution since 2015. Cisplatin concentrations in the perfusate were measured by inductively coupled plasma mass spectrometry (ICP-MS; PerkinElmer, America). The National Cancer Institute Common Terminology Criteria for Adverse Events (NCI-CTCAE) Version 4.0 was used to grade HIPEC-related adverse events (AEs) that presented within 3 weeks of HIPEC.

The Kolmogorov-Smirnov test was used to determine the distribution of continuous variables. Student’s *t* test was used to compare normally distributed continuous variables, whereas the Mann-Whitney *U* test was used to compare nonnormally distributed variables. The following equation was applied to calculate the quantity of cisplatin uptake during HIPEC:

$$ \mathrm{Actually}\ \mathrm{utilized}\ \mathrm{dose}\ \left(\mathrm{AD}\right)\ \left(\mathrm{mg}\right)=\mathrm{total}\ \mathrm{dose}\ \left(\mathrm{TD}\right)\ \left(\mathrm{mg}\right)-\mathrm{losing}\ \mathrm{dose}\ \left(\mathrm{LD}\right)\ \left(\mathrm{mg}\right) $$

$$ \mathrm{TD}=70\ \mathrm{mg}/{\mathrm{m}}^2\ast \mathrm{body}\ \mathrm{surface}\ \mathrm{area}\ \left({\mathrm{m}}^2\right)\ \left(\mathrm{the}\ \mathrm{resulting}\ \mathrm{TD}\ \mathrm{was}\ \mathrm{rounded}\ \mathrm{to}\ \mathrm{the}\ \mathrm{closest}\ \mathrm{integer}\right) $$

$$ \mathrm{LD}=\mathrm{volume}\ \left(\mathrm{ml}\right)\ \mathrm{of}\ \mathrm{the}\ \mathrm{perfusate}\ \left({\mathrm{VP}}_{\mathrm{retained}}\right)\ \mathrm{that}\ \mathrm{was}\ \mathrm{retained}\ \mathrm{in}\ \mathrm{the}\ \mathrm{treatment}\ \mathrm{system}\ \mathrm{at}\ \mathrm{the}\ \mathrm{end}\ \mathrm{of}\ \mathrm{HIPEC}\ast \mathrm{concentration}\ \mathrm{of}\ \mathrm{cisplatin}\ \mathrm{in}\ \mathrm{the}\ \mathrm{perfusate}\ \left(\mathrm{mg}/\mathrm{ml}\right). $$

To explore associations between the AD and potential clinical variables, linear regression analysis was conducted. These variables included patient age, body mass index (BMI), TD, VP_retained_ and serum levels of creatinine and albumin prior to HIPEC. Assumptions that underpin linear regression were confirmed as described previously. The final regression equation was established using a multiple linear regression model with the enter method. Variables with *P* values < 0.05 in the simple linear regression analysis were entered into the multiple linear regression analysis. Cox proportional hazards regression model was used to explore the prognostic influence of AD. Statistical tests were two-sided, and a *P* value < 0.05 was considered statistically significant. All data analyses were performed with SPSS 20.0 for Windows (SPSS, Inc., Chicago, IL).

## Results

A total of 62 patients were included in the final analysis. Table 1 summarizes the patient demographics and clinical characteristics. Because of massive ascites and pleural effusion, six patients had restricted activity (9.7%). Before HIPEC, abnormal serum creatinine levels were noted in one patient (1.6%) who had an elevated serum creatinine level (137 μmol/l) since diagnosis. This patient had no complaints and denied a history of kidney disease. Repeated creatinine tests and further kidney function evaluations were performed, but no evidence of acute or chronic kidney injury was identified. Then, the mean serum creatinine value (159 μmol/l) was used for the analysis. Of our cohort, 44 patients (71.0%) received HIPEC following PDS, while 18 patients (29.0%) received HIPEC following IDS.

**Table 1:** Demographic and clinical characteristics of patients

Characteristic	
Age (years), median (range)	51 (18, 76)
BMI (kg/m^2^), median (range)	22.1 (15.8, 34.8)
Body surface area (m^2^), median (range)	1.42 (1.21, 1.71)
ECOG performance status (%)	
Normal activity	56 (90.3)
Restricted activity	6 (9.7)
Histology (%)	
High grade serous adenocarcinoma	46 (74.2)
Mucinous adenocarcinoma	3 (4.8)
High grade endometrioid adenocarcinoma	3 (4.8)
Clear cell carcinoma	5 (8.1)
Malignant mixed mullerian tumor	5 (8.1)
FIGO stage (%)	
IIIC	43 (69.4)
IV	19 (30.7)
Surgical treatment (%)	
Primary debulking surgery	44 (71.0)
Interval debulking surgery	18 (29.0)
Gross residual disease (%)	
Yes	43 (69.4)
No	19 (30.7)
Surgical complexity (%)	
Low	5 (8.1)
Moderate	40 (64.5)
High	17 (27.4)
ICU stay (%)	
Yes	2 (3.2)
No	60 (96.8)
Serum creatinine (umol/L), median (range)	
Prior to HIPEC	63.5 (49, 159)
Following HIPEC	62.0 (48, 149)
Serum albumin (g/l), median (range)	
Prior to HIPEC	25 (15, 36)
Following HIPEC	22 (15, 30)

*BMI* Body mass index, *ECOG* Eastern cooperative oncology group, *HIPEC* Hyperthermic intraperitoneal chemotherapy, *ICU* Intensive care unit

Table 2 shows variables that were related to the cisplatin dose. The median TD and LD values were 95 mg (range: 85–120 mg) and 20.7 mg (range: 9.6–37.8 mg), respectively. Based on the formulation mentioned above, the median calculated AD value was 75.8 mg (range: 48.3–92.4 mg). The AD/TD ratio refers to the utility rate of cisplatin, and the median percentage was 79.2% (range: 62.2–90.4%).

**Table 2:** Hyperthermic intraperitoneal chemotherapy related parameters

Parameter	
TD (mg), median (range)	95 (85, 120)
Perfusate at the end of HIPEC	
Volume (ml), median (range)	1835 (1050, 2670)
Concentration of cisplatin (μg/ml), median (range)	11.3 (6.6, 19.4)
LD (mg), median (range)	20.7 (9.6, 37.8)
AD (mg), median (range)	75.8 (48.3, 92.4)
Utility rate of cisplatin^a^ (%), median (range)	79.2 (62.2, 90.4)

*AD* Actually utilized dose, *LD* Losing dose, *HIPEC* Hyperthermic intraperitoneal chemotherapy, *TD* Total dose;

^a^Utility rate of cisplatin = actually used dose of cisplatin (mg)/ total dose of cisplatin (mg)

**Fig. 1:**
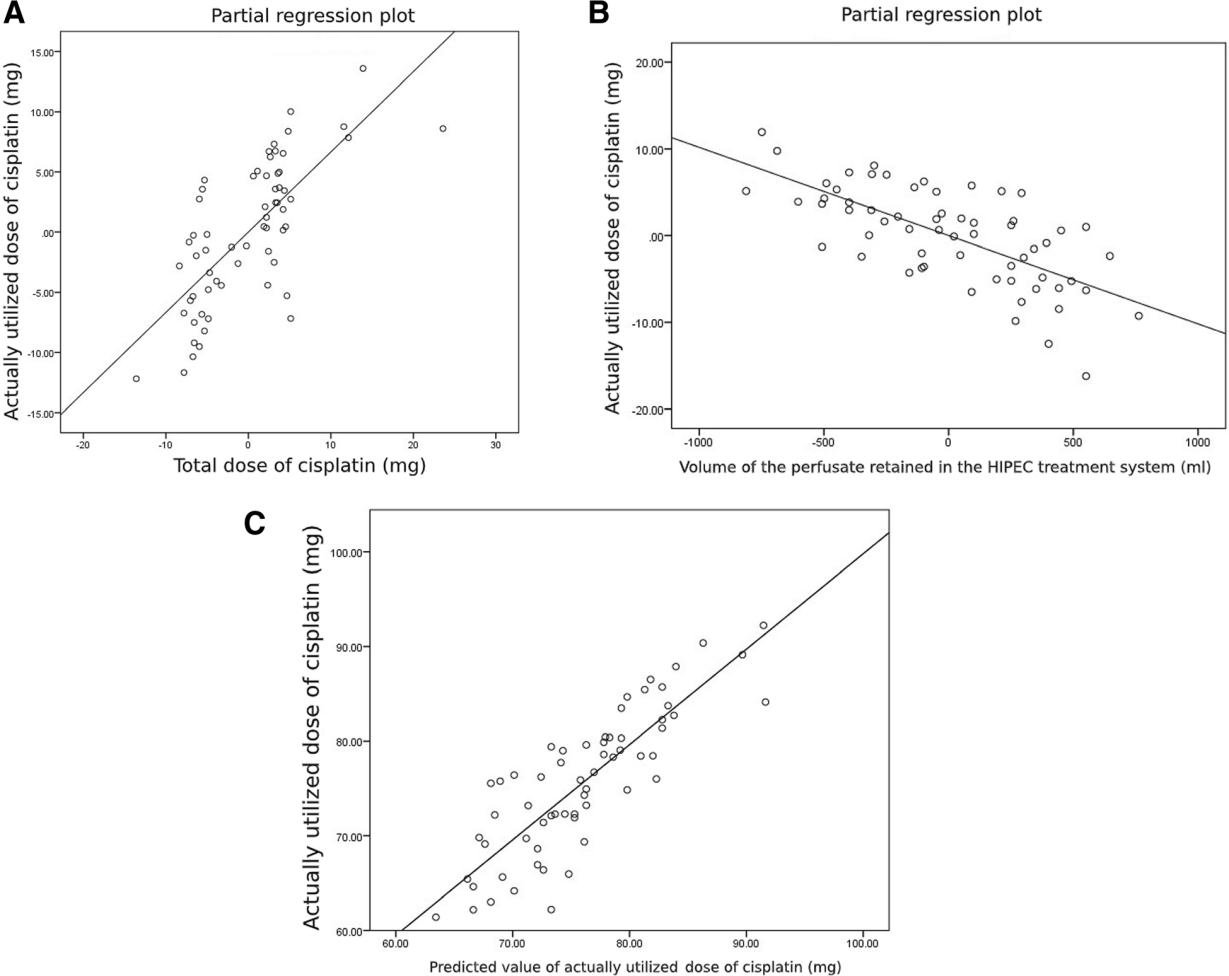
**a**. The linear relationship between actually utilized dose of cisplatin (mg) and total dose of cisplatin. **b**. The linear relationship between actually utilized dose of cisplatin (mg) and volume of the perfusate retained in the HIPEC treatment system (ml). **c**. The linear relationship between the observed value of actually utilized dose of cisplatin (mg) and the predicted value of actually utilized dose of cisplatin (mg). AD, actually utilized dose (mg); HIPEC, hyperthermic intraperitoneal chemotherapy; TD, total dose (mg); VP_retained_, volume of the perfusate that was retained in the treatment system at the end of hyperthermic intraperitoneal chemotherapy (ml)

The results of the linear regression analysis are summarized in Table 3. The TD and VP_retained_ had a linear relationship with the AD (Fig. 1a and b) and were able to significantly predict the AD on simple linear regression analysis. Based on these two factors, multiple linear regression analysis was conducted, and a significant regression equation was formulated [F (2, 59) = 71.419, *P* < 0.0001], with an *R*^2^ of 0.708. The formula was as follows: predicted AD (mg) = 30.079 + 0.667 TD (mg) – 0.010 VP_retained_ (ml). Therefore, when maintaining all other variables constant, the AD increased 0.667 mg for each milligram increase in the TD, while the AD decreased 0.010 mg for each milliliter increase in the VP_retained_. Both the TD and VP_retained_ were significant predictors for the AD. The TD and VP_retained_ accounted for 69.8% (adjusted *R*^2^ = 0.698) of the explained variability in the AD. Figure 1c illustrates scatter plots of the actual vs predicted AD values.

**Table 3:** Linear regression model results for actually utilized dose of cisplatin

	β	95% CI for β	*P* value	β	95% CI for β	*P* value
Age (years)	−0.012	−0.169, 0.146	0.881	–	–	–
BMI (kg/m^2^)	−0.117	−0.705, 0.472	0.693	–	–	–
Serum creatinine prior to HIPEC (umol/l)	0.013	−0.103, 0.129	0.822	–	–	–
Serum albumin (g/l) prior to HIPEC	0.078	−0.308, 0.464	0.689	–	–	–
TD (mg)	0.777	0.554, 1.000	< 0.001	0.667	0.500, 0.833	< 0.001
V_Pretained_ (ml)	−0.012	−0.016, − 0.008	< 0.001	0.010	−0.013, − 0.007	< 0.001

*BMI* Body mass index, *HIPEC* Hyperthermic intraperitoneal chemotherapy, *TD* Total dose, *V*_*Pretained*_ Volume (ml) of the perfusate that was retained in the treatment system at the end of hyperthermic intraperitoneal chemotherapy;

HIPEC-related AEs are summarized in supplementary Table 1. Following HIPEC, the most common AE was abdominal pain/distention, which was noted in ten patients (16.1%). Four patients (6.5%) reported AEs of grade 3, including 2 cases of neutropenia, one case of abdominal pain, and one case of vomiting. No patient developed AEs of grade 4.

Within a median follow-up time of 23 months (range: 9–35 months), recurrence was noted in 35 patients (56.4%), and the median recurrence-free survival (PFS) time was 16 months (range: 4–29 months). Ten patients (16.1%) died of disease, and the median overall survival (OS) time was not reached. In Cox regression analysis, we did not find that AD was associated with PFS [hazard ratio (HR) = 1.02, 95% confidence interval (CI): 0.98–1.06; *P* = 0.428] or OS (HR = 1.01, 95% CI: 0.95–1.07; *P* = 0.813).

## Discussion

HIPEC has evolved over three decades and cisplatin is among the first-line drugs. However, there is no clearly defined standardization of regimen for cisplatin in the hyperthermic setting, and the reported dose in previous studies varies significantly, from 15 mg/m^2^ to 250 mg/m^2^ [13, 18, 19]. The most accepted cisplatin based HIPEC regimens in literature are the “Sugarbaker Regimen” [add cisplatin (50 mg/m^2^) + doxorubicin (15 mg/m^2^) to 2 L 1.5% dextrose peritoneal dialysis solution; treatment duration: 90 min] [20] and the “National Cancer Institute Milan Regimen” [15.25 mg/L of doxorubicin and 43 mg/L of cisplatin for 90-min HIPEC treatment; chemotherapy solution 4–6 L based on capacity of the peritoneal space] [13, 21]. Despite of this, neither of them has been recommended in current guidelines or validated in gynecologic cancer patients. Most of the side effects associated with hyperthermic cisplatin are dose dependent [22], so the lack of a standard dose regimen represents a notable challenge for the safe application of HIPEC. During HIPEC, the TD of cisplatin can be divided into two portions: one portion (AD) is directly absorbed or retained in the peritoneal cavity, while the other portion (LD) is retained in the perfusate and exits the body at the end of treatment. The variable AD is critical since it enables physicians to know the uptake quantity of cisplatin and thus can be used to tailor dose regimens. In the present study, we found that the AD is significantly correlated with the TD and VP_retained_. Based on these two factors, an equation was established with an adjusted *R*^2^ = 0.698, which suggested a moderate ability to predict the AD. In addition, we did not find that AD had prognostic impact.

The pharmacokinetics of cisplatin under hyperthermic conditions have been investigated previously [14–16]. Cashin’s study included ten patients who received HIPEC for peritoneal surface malignancies [14]. A combination of cisplatin (50 mg/m^2^) and doxorubicin (15 mg/m^2^) was added to the perfusate, which was administered at 41.1–43 °C for a duration of 90 min. The reported mean half-life (t1/2) of perfusate cisplatin was 18.4 min. Accordingly, the authors concluded that after 75 min, there is little active cisplatin left in the perfusate. In another study by Ansaloni et al., 13 ovarian cancer patients received HIPEC, and a combination of cisplatin (100 mg/m^2^) and paclitaxel (175 mg/m^2^) was given at 41–43 °C [15]. Similarly, the authors reported that both drugs could be rapidly taken up from the perfusate by peritoneal tissue and the absorption of cisplatin could not be influenced by lowering the time of perfusion to 60 min. The present study is retrospective, so it is impossible for us to get patients’ blood sample and carry out pharmacokinetic assessment. Given the importance of the amount of cisplatin actually utilized in the living body, we calculated the utility rate of cisplatin using AD/TD ratio, which could be considered an indirect pharmacokinetic parameter. The median cisplatin utility rate in our cohort was 79.2% (range: 62.2–90.4%); therefore, approximately 80% of cisplatin was utilized after 60 min of HIPEC. This result indicates efficient uptake of cisplatin during HIPEC, which is consistent with previous reports [14, 15]. Loss of cisplatin during HIPEC is inevitable. Given our findings and current evidence, the necessity of minimizing the loss by methods such as flushing the device at the end of HIPEC is worth investigation in future studies.

In the NCCN ovarian cancer guidelines, the recommended dose for hyperthermic cisplatin is 100 mg/m^2^ [9]. This recommendation is based on the phase III OVHIPEC trial [4], where 245 patients were randomized to receive IDS either with or without HIPEC. The authors reported that the addition of HIPEC did not increase the risk of AEs. However, the administration of cisplatin in this trial was according to the following schedule: 50% of the dose at start, 25% at 30 min and 25% at 60 min. In this way, the maximum dose in the abdomen was lower than 100 mg/m^2^. An additional concern is that information on the influence of HIPEC on renal function was not detailed. Therefore, it remains unknown whether a dose of 100 mg/m^2^ of cisplatin is safe for HIPEC. In the present study, all patients received a dose of 70 mg/m^2^ cisplatin. The rationality of this dose regimen has been validated in Gouy’s study [23]. In the phase I dose escalation trial, four dose levels were planned for cisplatin: 50, 60, 70, and 80 mg/m^2^. The observed grade 4 dose-limiting toxicity was renal insufficiency, which did not occur until cisplatin was administered at dose level 4 (80 mg/m^2^). Because several patients developed prolonged renal function impairment, the authors recommended a 70 mg/m^2^ dose of cisplatin for HIPEC. In the current cohort, no patients developed kidney dysfunction following HIPEC, which is in line with our previous findings and confirms Gouy’s conclusion [23].

HIPEC-related kidney injury has been a clinical concern. The reported incidence of major renal toxicity ranges from 1.3 to 5.7% [24–26]. Among previous studies regarding this issue, Cata’s study, which retrospectively reviewed 475 patients, has the largest sample size examined to date [27]. The authors reported that acute kidney injury (AKI) following HIPEC was independently associated with patient age, body mass index, the use of cisplatin or oxaliplatin as the agent, the preoperative administration of pregabalin and estimated blood loss [27]. In addition, the risk of kidney dysfunction is also characterized by ethnical differences [28]. For Chinese ovarian cancer patients, Sin et al. identified predictors for AKI following HIPEC that included age, baseline levels of creatinine, the estimated glomerular filtration rate and albumin, the number of cycles of preoperative carboplatin, the time interval between NACT and debulking surgery, and the volume of blood transfusions [29]. Of note, Sin et al. reported that 9.4% of their patients developed NCI-CTCAE grade 3 and 4 renal impairment, and 5.7% of their patients needed renal replacement therapy [29]. The incidence reported in this study is much higher than that reported in other studies [24–26], and a possible explanation is that many patients received cycles of platinum-based chemotherapy before HIPEC, and a high dose of cisplatin (90 mg/m^2^) was prescribed in HIPEC. Although an even higher dose of cisplatin (100 mg/m^2^) was used in OVHIPEC trial, some aspects of the trial are questionable, which has been discussed above. Besides, all patients in this trial received sodium thiosulphate and adequate hydration. Appropriate use of these measures can effectively prevent nephrotoxicity which could also provide additional explanation for why such high dose of hyperthermic cisplatin did not result in an increased incidence of kidney injury [30]. Collectively, current evidence suggests that HIPEC-related nephrotoxicity is complex and can be affected by many factors. The selection of patients based on risk stratification, optimized cisplatin dose regimens and appropriate protective measures can help reduce the risk of HIPEC-related nephrotoxicity.

The treatment temperature in our study was 43 ± 0.01 °C, which is in accordance with the CACA guidelines [10]. This recommendation is based on the evidence that the synergistic effect of hyperthermia and cisplatin can be dramatically increased at 42 °C [10]. Data from clinical research also confirmed the safety and effectiveness of HIPEC at 43 °C [31–34]. In general, the properties of biological tissues, the toxicity of drugs and their mutual influences change under hyperthermic conditions; some of the changes are temperature dependent [35]. Therefore, it is necessary to explore drug uptake in the hyperthermic setting at a given temperature. In addition, other parameters, including flow rate during HIPEC and perfusate dwelling time, have potential impact on cisplatin uptake. However, these data were not available in the present study; their influence is worthy of further studies.

The present study provides the first tool that can be used to calculate the AD of cisplatin during HIPEC, which makes it possible to further explore and determine whether a supplementation on cisplatin is necessary in the clinical setting. Of note, AD can be influenced by many factors. However, since the design of present study is retrospective, the omission of potential factors is inevitable, which certainly adversely affects the accuracy of the predictive equation. In addition, the adjusted R^2^ value of the predictive equation was 0.698 indicating that predictive performance has not yet reached a satisfying level. Given these limitations, we must acknowledge that the prediction formula still needs to be refined and more trials are needed to validate its performance. In addition, HIPEC is recommended as an adjuvant therapy following cytoreduction in the CACA guidelines [10]; however, since all patients in the present study were retrospectively reviewed, we cannot reasonably explain how patients were selected to receive cytoreduction plus HIPEC rather than cytoreduction alone. Another limitation is that not all patients received the same comprehensive treatment model, and the follow-up period was short. Therefore, although the recurrence-free survival of our cohort is comparable to that in previous studies, the median OS was not achieved. Additionally, the sample size of the present study was relatively small, and the potential influence from the type of HIPEC device cannot be excluded.

## Conclusions

For ovarian cancer patients who receive cisplatin for HIPEC at 43 °C, the AD of cisplatin can be calculated from the TD and VP_retained_ using the following equation: AD (mg) = 30.079 + 0.667 TD (mg) – 0.010 VP_retained_ (ml). AD has no prognostic influence. Our work could help guide dose planning for cisplatin when HIPEC is indicated. Larger prospective trials are needed to validate our findings.

## Supplementary Information

**Additional file 1.** Adverse events.

## Data Availability

The datasets generated during and analyzed during the current study are available from the corresponding author on reasonable request.

## Abbreviations

AD: Actually utilized dose
AKI: Acute kidney injury
AE: Adverse event
BMI: Body mass index
CI: Confidence interval
HR: Hazard ratio
HIPEC: Hyperthermic intraperitoneal chemotherapy
ICP-MS: Inductively coupled plasma mass spectrometry
IDS: Interval debulking surgery
IPC: Intraperitoneal chemotherapy
LD: Losing dose
NCCN: National Comprehensive Cancer Network
NACT: Neoadjuvant chemotherapy
OS: Overall survival
PDS: Primary debulking surgery
PFS: Recurrence-free survival
CACA: The China Anti-Cancer Association
NCI-CTCAE: The National Cancer Institute Common Terminology Criteria for Adverse Events
TD: Total dose
Vpretained: Volume of the perfusate that was retained
