# Supplementary material for: Calculating the dose of cisplatin that is actually utilized in hyperthermic intraperitoneal chemotherapy among ovarian cancer patients
Source: J Ovarian Res. 2021 Jan 8;14:9. doi: 10.1186/s13048-021-00764-6 (PMC7796576; doi:10.1186/s13048-021-00764-6)
Supplement: Supplementary file 1 — Additional file 1. Adverse events. [file 13048_2021_764_MOESM1_ESM.docx]

Supplementary 1. Adverse events

| Adverse event | NCI-CTCAE 4.0 | | | |
| --- | --- | --- | --- | --- |
|  | Grade 1 | Grade 2 | Grade 3 | Grade4 |
| Neutropenia | 2 | 4 | 2 | 0 |
| Abdominal pain/distention | 7 | 2 | 1 | 0 |
| Diarrhea | 2 | 0 | 0 | 0 |
| Dyspnea | 0 | 1 | 0 | 0 |
| Vomiting | 0 | 1 | 1 | 0 |
| Gastrointestinal anastomotic leak | 0 | 0 | 0 | 0 |
| Febrile neutropenia | 0 | 0 | 0 | 0 |
| Renal | 0 | 0 | 0 | 0 |
| Infection | 0 | 0 | 0 | 0 |

NCI-CTCAE, the national cancer institute common terminology criteria for adverse events;
